# Supplementary material for: All-dielectric scale invariant waveguide
Source: Nat Commun. 2023 Oct 21;14:6675. doi: 10.1038/s41467-023-42234-1 (PMC10590422; doi:10.1038/s41467-023-42234-1)
Supplement: Supplementary file 1 — Supplementary Information [file 41467_2023_42234_MOESM1_ESM.pdf]

Supplementary Material for All-dielectric Scale Invariant Waveguide

Janderson R. Rodrigues, Utsav D. Dave, Aseema Mohanty¸ Xingchen Ji, Ipshita Datta, Shriddha Chaitanya, Euijae Shim, Ricardo Gutierrez-Jauregui, Vilson R. Almeida, Ana Asenjo-Garcia, Michal Lipson

Table of Contents

1. Electromagnetic description of the device
2. Ray-optics description of the device
3. Comparison with a slot-waveguide
4. Effect of material losses on the device
5. Analytical-based method to design the 2D devices
6. Scale-invariant effect in low-refractive-index contrast waveguides
7. Materials characterization and the waveguide design
8. Analysis of the operation bandwidth of the device
9. Effect of geometric imperfections on the device
10. Lensed-fiber pre-characterization

References

1- Electromagnetic description of the device

Without loss of generality, we develop analytical models by considering a slab (planar) waveguide structure shown schematically in Fig. 1(a) in the main text. The dielectric structure is formed by two planar waveguides of high refractive index material (n_H) with thickness $t/2$, separated by an intermediary-index material (n_S) with thickness d, and surrounded by a low-refractive index material (n_L). The waveguides are symmetrical in both the x- and y-direction. We assume that the waveguides are infinite in extent in the x-direction, translational invariance of the structure in the propagation direction z. We also assume that the planar waveguide is excited by a harmonic source with a fixed wavelength λ_0 and a vacuum wavevector of magnitude k_0, where $k_0 = 2\pi/\lambda_0$. Maxwell's equations, under these assumptions, lead to two independent sets of solutions for the field polarization: Transverse Electric (TE) or Transverse Magnetic (TM), depending on the orientation of the main component of the electric field. In the TE case, the electric field is polarized along the x-axis (E_x, H_y, H_z), and in the TM counterpart the transverse component of the electric field is polarized along the y-axis (E_y, E_z, H_x).

The $E_x(y)$ component of TE polarization is given by solving the following transverse wave equation,

$$\frac{\partial^2 E_x(y)}{\partial y^2} + {k_0}^2\left({n_{sf}}^2(y) - {n_{eff}}^2\right)E_x(y) = 0 \qquad \text{S1}$$

where n_{eff} is the mode's effective refractive index. The vacuum (or free space) wavevector magnitude k_0 is related to the angular frequency ω_0 and the speed of light in vacuum c_0 through $k_0 = \omega_0/c_0 = \omega_0\sqrt{\mu_0\varepsilon_0}$, where ε_0 and μ_0 are the vacuum electric permittivity and the magnetic permeability, respectively. The refractive index $n_{sf}(y)$ is a step-index function, which is equal to n_S, for $|y| < {}^{d}/_{2}$, to n_H for ${}^{d}/_{2} \le |y| \le {}^{d}/_{2} + {}^{t}/_{2}$, and to n_L $|y| > {}^{d}/_{2} + {}^{t}/_{2}$.

In the TE polarization, the spatial-distribution components of the fields are related to each other through:

$$\left[E_x(y)\ H_y(y)\ H_z(y)\right]^T = \left[1 \quad -\frac{k_0 n_{eff}}{\omega_0\mu_0} \quad \frac{i}{\omega_0\mu_0}\frac{\partial}{\partial y}\right]^T E_x(y)\,, \qquad \text{S2}$$

where $[\cdot]^T$ denotes matrix transpose.

Similarly, the $H_x(y)$ component of TM polarization is given by solving the following equation,

$$\frac{\partial^2 H_x(y)}{\partial y^2} + k_o{}^2\left(n_{sf}{}^2(y) - n_{eff}{}^2\right)H_x(y) = 0 \qquad \text{S3}$$

where the spatial-distribution components are given by:

$$\left[E_y(y)\ E_z(y)\ H_x(y)\right]^T = \left[\frac{1}{n_{sf}{}^2(y)}\frac{n_{eff}}{c_0\varepsilon_0} \quad \frac{-i}{n_{sf}{}^2(y)}\frac{1}{\omega_0\varepsilon_0}\frac{\partial}{\partial y} \quad 1\right]^T H_x(y)\ , \qquad \text{S4}$$

Due to the structure symmetry, with respect to the y-axis, the electromagnetic field distributions for each mode can be classified into symmetric/even or antisymmetric/odd. The transcendental (characteristic) equations of the symmetric eigenmodes for both the TE and TM polarizations are given, respectively, by

$$\tanh\left(k_s\frac{d}{2}\right) = \frac{\kappa_H{}^2\tan\left(\kappa_H\frac{t}{2}\right) - \kappa_H k_L}{k_L k_S\tan\left(\kappa_H\frac{t}{2}\right) + \kappa_H k_S} \qquad \text{S5}$$

and

$$\tanh\left(k_s\frac{d}{2}\right) = \frac{\frac{\kappa_H{}^2}{n_H{}^4}\tan\left(\kappa_H\frac{t}{2}\right) - \frac{\kappa_H}{n_H{}^2}\frac{k_L}{n_L{}^2}}{\frac{k_L}{n_L{}^2}\frac{k_S}{n_S{}^2}\tan\left(\kappa_H\frac{t}{2}\right) + \frac{\kappa_H}{n_H{}^2}\frac{k_S}{n_S{}^2}} \qquad \text{S6}$$

where κ_H, k_L, k_S are the transversal wavevector, and the field decay coefficients, respectively given by:

$$\kappa_H = k_0\sqrt{n_H{}^2 - n_{eff}{}^2}, \qquad \text{S7}$$

$$k_L = k_0\sqrt{n_{eff}{}^2 - n_L{}^2}, \qquad \text{S8}$$

and

$$k_S = k_0\sqrt{n_{eff}{}^2 - n_S{}^2} \qquad \text{S9}$$

The numerical solutions of Equations S5 and S6 yield the eigenvalues of n_{eff} that correspond to allowed guided modes in the waveguide for each polarization. For these modes, the universal guiding condition $n_S < n_{eff} < n_H$ must be satisfied, assuming $n_H > n_S \geq n_L$. However, here we show next that this condition can be relaxed for symmetric structures by analyzing that case where $n_{eff} = n_S$ and $n_{eff} < n_S$, which results in unusual modal properties.

The solutions of Eq. S1 and Eq. S3 are obtained by applying the following boundary conditions: the continuity of the tangential electric and magnetic fields (and their derivatives) at the dielectric interfaces $\left(x = \left|{}^{d}/_{2}\right| \text{ and } x = \left|{}^{d}/_{2} + {}^{t}/_{2}\right|\right)$ and finite energy requirements in the field spatial distributions, Equations S2 and S4. Then, the $E_x(y)$ and $E_y(y)$ components for the TE and TM polarizations, respectively, can be expressed as:

$$E_x(y) = E_0 \begin{cases} \cosh(k_S y), \quad |y| \leq {}^{d}/_{2} \\ \cosh\left(k_S\, {}^{d}/_{2}\right) \cos\left[\kappa_H\left(|y| - {}^{d}/_{2}\right)\right] + \\ + \dfrac{k_S}{\kappa_H} \sinh\left(k_S\, {}^{d}/_{2}\right) \sin\left[\kappa_H\left(|y| - {}^{d}/_{2}\right)\right], \qquad {}^{d}/_{2} \leq |y| \leq {}^{d}/_{2} + {}^{t}/_{2} \\ \left\{\cosh\left(k_S\, {}^{d}/_{2}\right) \cos\left(\kappa_H\, {}^{t}/_{2}\right) + \right. \\ \left. + \dfrac{k_S}{\kappa_H} \sinh\left(k_S\, {}^{d}/_{2}\right) \sin\left(\kappa_H\, {}^{t}/_{2}\right)\right\} e^{-k_L(y-t)}, \qquad |y| \geq {}^{d}/_{2} + t \end{cases} \qquad \text{S10}$$

and

$$E_y(y) = \frac{E_0 n_{eff}}{c_0 \varepsilon_0} \begin{cases} \dfrac{1}{{n_S}^2} \cosh(k_S y), \quad |y| \leq {}^{d}/_{2} \\ \dfrac{1}{{n_H}^2} \cosh\left(k_S\, {}^{d}/_{2}\right) \cos\left[\kappa_H\left(|y| - {}^{d}/_{2}\right)\right] + \\ + \dfrac{1}{\kappa_H} \dfrac{k_S}{{n_S}^2} \sinh\left(k_S\, {}^{d}/_{2}\right) \sin\left[\kappa_H\left(|y| - {}^{d}/_{2}\right)\right], {}^{d}/_{2} \leq |y| \leq {}^{d}/_{2} + {}^{t}/_{2} \\ \dfrac{1}{{n_C}^2} \left\{\cosh\left(k_S\, {}^{d}/_{2}\right) \cos\left(\kappa_H\, {}^{t}/_{2}\right) + \right. \\ \left. + \dfrac{{n_H}^2}{\kappa_H} \dfrac{k_S}{{n_S}^2} \sinh\left(k_S\, {}^{d}/_{2}\right) \sin\left(\kappa_H\, {}^{t}/_{2}\right)\right\} e^{-k_L(y-{}^{t}/_{2})}, \qquad |y| \geq {}^{d}/_{2} + {}^{t}/_{2} \end{cases} \qquad \text{S11}$$

where E_0 is the field amplitude.

At the critical point $n_{eff} = n_S$ leads $k_S = 0$ according to Eq. S9. Substituting this condition in the TE and TM transcendental equations (Eq. S5 and Eq. S6):

$$\tan\left(\kappa_H \frac{t}{2}\right) = \frac{k_L}{\kappa_H} \qquad \text{S12}$$

and

$$\tan\left(\kappa_H \frac{t}{2}\right) = \frac{{n_H}^2}{{n_L}^2}\frac{k_L}{\kappa_H} \qquad \text{S13}$$

which shows that the eigenvalues at the critical point do not depend on the middle-layer size, since these equations are independent of d. Besides that, one can notice that these equations are the transcendental equations of the single-layer symmetric waveguides, formed by the condition when the middle-layer size is equal to zero. Therefore, the critical point for a slab structure can be easily achieved by matching the effective index of the mode of the single slab waveguide (n_{eff}) with the refractive index of the material of the middle-layer (n_S).

On the other hand, the critical condition ($k_S = 0$) in field distributions (Eq. S10 and Eq.S11) leads to,

$$E_x(y) = \begin{cases} E_0, & |y| \leq {}^{d}/_{2} \\ E_0 \cos\left[\kappa_H\left(|y| - {}^{d}/_{2}\right)\right], & {}^{d}/_{2} \leq |y| \leq {}^{d}/_{2} + {}^{t}/_{2} \\ E_0 \cos\left(\kappa_H\, {}^{t}/_{2}\right) e^{-k_L\left(y - {}^{t}/_{2}\right)}, & |y| \geq {}^{d}/_{2} + {}^{t}/_{2} \end{cases} \qquad \text{S13}$$

for the TE polarization, and

$$E_y(y) = \begin{cases} \frac{1}{{n_S}^2} E_0, & |y| \leq {}^{d}/_{2} \\ \frac{1}{{n_H}^2} E_0 \cos\left[\kappa_H\left(|y| - {}^{d}/_{2}\right)\right], & {}^{d}/_{2} \leq |y| \leq {}^{d}/_{2} + {}^{t}/_{2} \\ \frac{1}{{n_L}^2} E_0 \cos\left(\kappa_H\, {}^{t}/_{2}\right) e^{-k_L\left(y - {}^{t}/_{2}\right)}, & |y| \geq {}^{d}/_{2} + {}^{t}/_{2} \end{cases} \qquad \text{S13}$$

for the TM polarization. From Equations S12 and S13, one can see that the TE and TM field distributions are uniform inside the middle-layer region at the critical point, with magnitude E_0 and ${n_H}^2/{n_S}^2\,E_0$, respectively. Besides that, they take the shape of the field distribution of a single symmetric waveguide scaled by the size of the middle layer's region d.

Another way of seeing this effect is by analyzing the wave equation (Eq. S1) in the middle layer's region $|y| < {}^{d}/_{2}$, which is given by

$$\frac{\partial^2 E_x(y)}{\partial y^2} - {k_S}^2 E_x(y) = 0 \qquad \text{S14}$$

At the critical point, it becomes

$$\frac{\partial^2 E_x(y)}{\partial y^2} = 0 \qquad \text{S15}$$

which leads to a linear solution of the kind

$$E_x(y) = \frac{dE_x(y)}{dy}\,y + E_0 = -i\omega_0\mu_0 H_z(y) + E_0 \qquad \text{S16}$$

Similarly, for the TM polarization

$$E_y(y) = \frac{dE_y(y)}{dy}\,y + E_0 = i\left({}^{\omega_0}/_{c_0}\right) n_{eff} E_z(y) + E_0 \qquad \text{S17}$$

Therefore, if a middle layer, in which the refractive index matches the critical condition, is included at the maximum of the electromagnetic field, it has a uniform distribution in that region. Near this condition, it presents a linear solution with a given inclination. Besides that, this feature allows the expansion of the effect to high-order mode. We also noticed that this effect is similar to the epsilon-near-zero (ENZ) effect; however, in this case, it happens for pure dielectric materials and is oriented in the transverse direction, instead of being in the propagation direction as in the case of an ENZ material.

In another description, the field inside the middle layer can be described by a superposition of two opposite propagating waves, which can be decomposed as $E_x(y) = E_0 \cosh(k_S y) = E_0/2\,(e^{+k_S y} + e^{-k_S y})$. Following this analysis, the symmetric slab waveguide structure can also be decomposed into two mirror images of a standard asymmetric slab waveguide, as shown in Fig. S1(a).

Asymmetric waveguide left + Asymmetric waveguide right = Symmetric waveguide

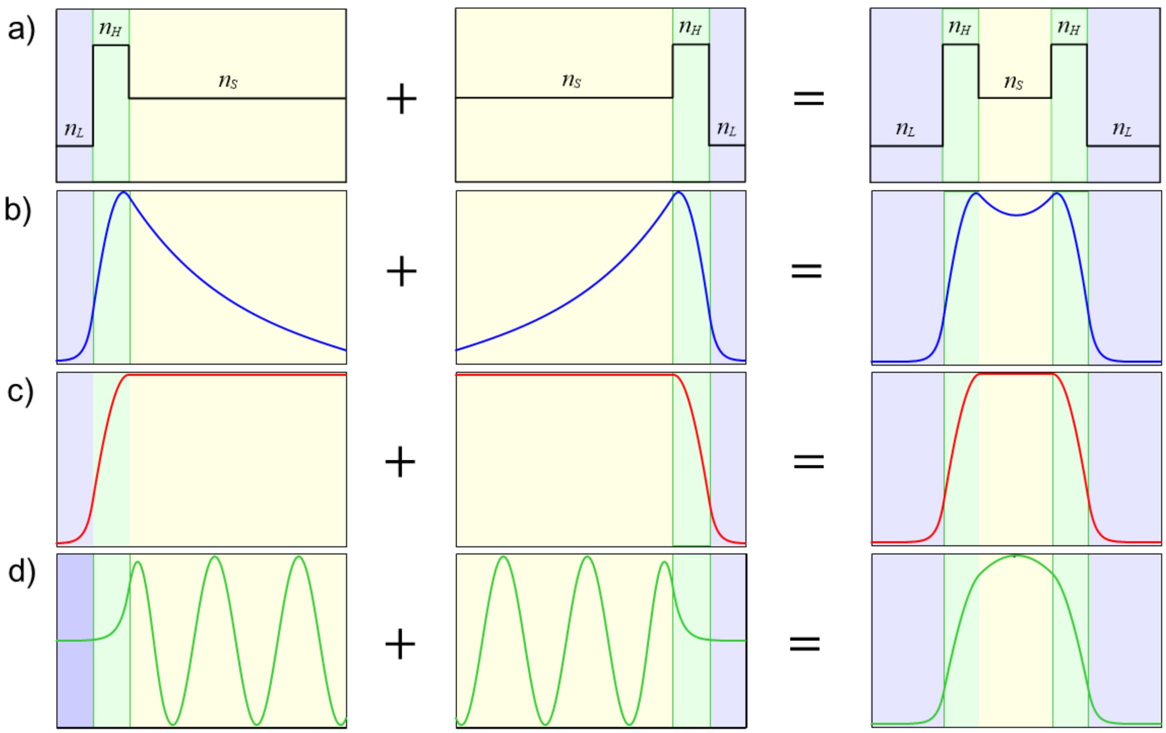

Figure S1. Decomposing of the symmetric waveguide structure into two mirror-image asymmetric waveguides. (a) Refractive index profiles of two asymmetric waveguides are spatially oriented in opposition to each other, followed by an index profile of a symmetric waveguide created by the spatial composition of the previous two, where the index relation $n_H > n_S > n_L$ is always preserved. **(b)** For k_S real, the field represents the usual evanescent field decay outside of the waveguide core. The symmetric waveguide behaves as a conventional coupled waveguide with a Gaussian-like distribution, where most of the light is concentrated in the highest index material and decay profile between and outside them. **(c)** The critical point, where $k_S = 0$, corresponds to the cut-off condition of the asymmetric waveguides and their field distribution diverges. In contrast, the symmetry of the symmetric waveguide ensures the stability of the optical mode at this point and creates this uniform non-Gaussian profile inside the middle layer. **(d)** For imaginary k_S, the electromagnetic distributions show periodic oscillatory behavior characteristics of substrate radiation modes. However, on the symmetric waveguide, the counter-propagating oscillatory fields constructively interfere creating lossless (internal) radiation modes $n_{eff} < n_S$, where most of the light is concentrated in the middle material. The mirror-symmetry provides access to these previously inaccessible regimes.

The field decay length τ_S in the n_S-material is defined by $\tau_S = 1/k_S$ and determines the distance the maximum field amplitude in that region decay by (1/e ≈ 37%) as shown in Fig. S1(b). By analyzing the critical point, where k_S vanishes the field decay length diverges to infinite, as presented in Fig. S1(c). This point corresponds to the cut-off frequency of the asymmetric waveguides. In the mirror-symmetric structure, this singularity is responsible for the scale invariance of the mode in relation to the size of the middle layer. For k_S imaginary the field becomes radiative and therefore extremely lossy. In contrast, in the new structure, the radiative field remains confined to the middle material n_S, preventing radiation loss.

Figure S2 shows examples of numerical simulation of the scale-invariant waveguide at the critical point for the fundamental modes of the TE and TM polarizations. The simulations for the third-order modes are shown in Fig. S3.

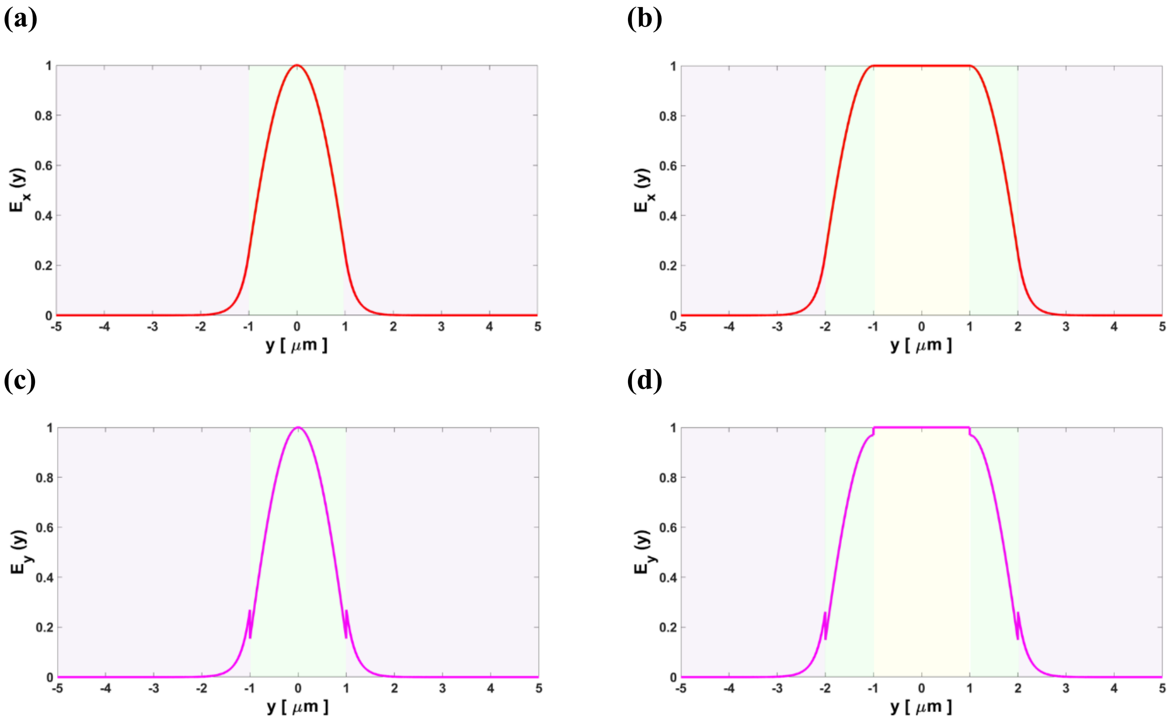

Figure S2. Fundamental modes simulation results for the scale invariant waveguide for TE and TM polarization. (a-b) Fundamental TE mode for $d = 0$ and 2 μm. **(c-d)** Fundamental TM mode for $d = 0$ and 2 μm.

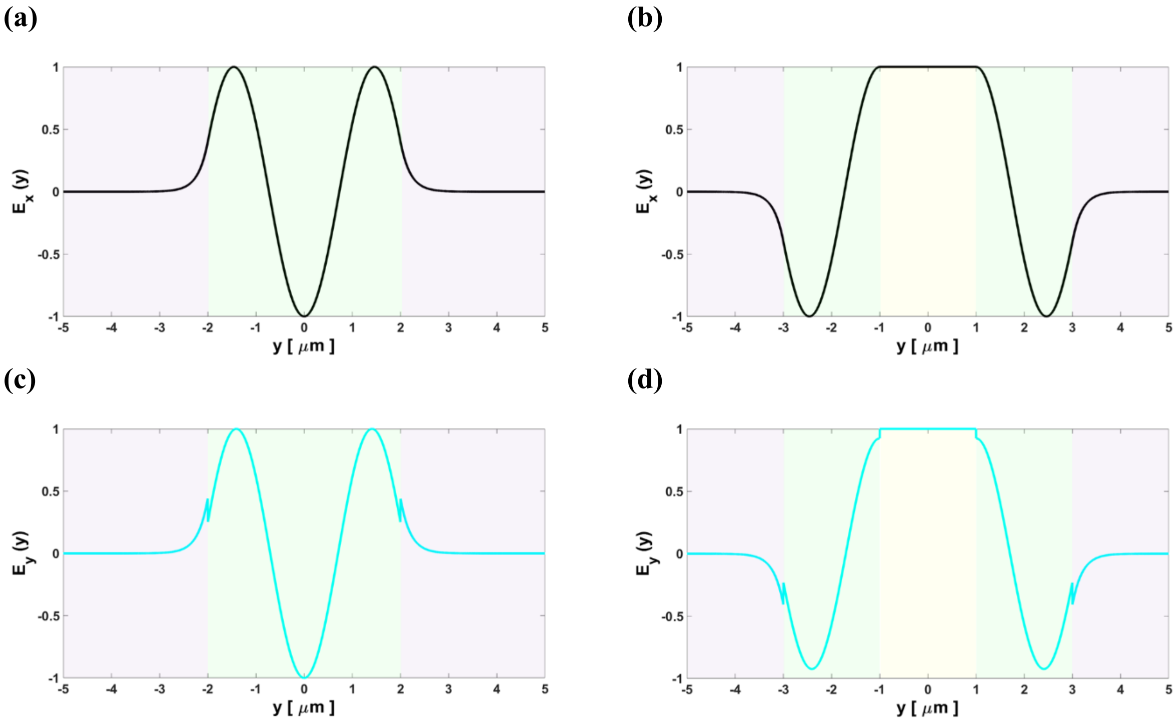

Figure S3. High-order modes simulation results for the scale invariant waveguide for TE and TM polarization. (a-b) 3rd order TE mode (TE_2) for d = 0 and $d = 2\ \mu m$. **(c-d)** 3rd order TM mode (TM_2) for $d = 0$ and 2 μm.

2- Ray-optics description of the device

We employ ray-optics concepts to show that the guiding mechanisms emerge at the critical angle and below it. The ray-optics schematics of each case are shown on the zoom extents presented in the right column of Figures S4(b-d), where the angle of incidence, θ_{inc}, and the critical angle, θ_{crit}, are defined at the interface between the core (n_H) and middle-layer (n_S). When $\theta_{inc} > \theta_{crit}$, the light rays undergo TIR. At the critical point $\theta_{inc} = \theta_{crit}$, the refractive waves travel at the interfaces between the two materials. Finally, when $\theta_{inc} < \theta_{crit}$, the reflections at the interface are only partial with refracted rays extending to the substrate. are only partial with refracted rays extending to the substrate creating a loss. On the other hand, in the symmetric waveguide structure, these refracted waves are trapped inside the middle layer.

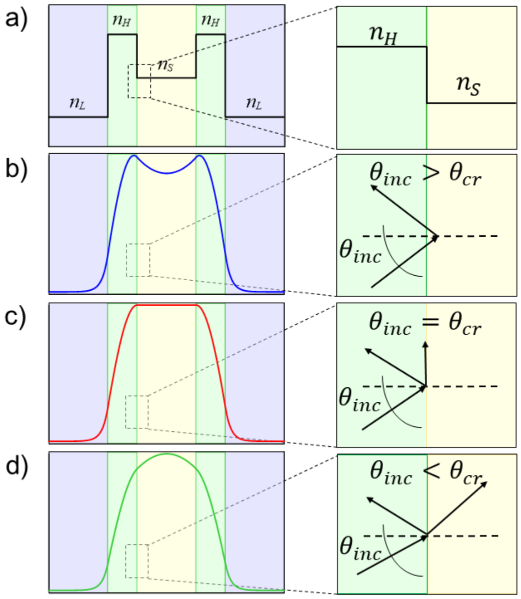

Figure S4. Guiding light at the critical angle and beyond. (a) Ray-optics schematics and electromagnetic spatial distributions of the electric field (E(y)) for fundamental TE mode, for different values of the angle of incidence, θ_{inc}, at the interfaces between the two highest index materials, (n_H/n_S). **(b)** When $\theta_{inc} > \theta_{cric}$, the light rays undergo through total internal reflection (TIR) at the interface and the field distributions generate evanescent waves, with exponential fields decaying into the substrate. **(c)** When $\theta_{inc} = \theta_{cric}$ the refractive waves travel at the interfaces. **(d)** Finally, when $\theta_{inc} < \theta_{crit}$ the reflections are only partials and they are accompanied by refractions to the substrate **(e)**.

3- Comparison with a slot-waveguide

The slot waveguide is one the most used photonic waveguides to enhance the light-matter interaction with low-index materials. Here, we compare the properties of the proposed waveguide with those of a slot waveguide composed of the same materials. First, we design a scale-invariant waveguide formed by two high-index-material slabs $n_H = 3.5$ with thickness $t = 130\,nm$, separated by an intermediary-index material $n_S = 2.4212$ of thickness $d = 0.05\,\lambda$ and $d = 0.35\,\lambda$, respectively, surrounded by a low-index-material $n_L = 1.5$. The slabs thicknesses were designed in such a way that the TM fundamental mode operates at the critical point at the wavelength of $\lambda_0 = 1.55\,\mu m$, shown in Fig. S5(a-b). Then, we consider conventional Slot-waveguide dimensions, with slab thicknesses of $t = 400\,nm$, illustrated in Fig. S2(c-d). For the sake of completeness, in Fig. S5 (e-f), we also consider a single slab waveguide composed of a core material with refractive-index $n_H = n_S = 2.4212$ and thickness $t = d$, also surrounded by the low-index material.

In Figs. S6 we show the computed values for the confinement factor for power (solid lines) and electric field intensities (dashed lines) inside the middle layer's region (n_S) as a function of its thickness, d, normalized by the wavelength λ_0. Figure S1(a) shows that the scale-invariant presents a huge improvement in both the power and intensities confinement factors inside the middle layer. Such behavior can be understood by comparing the distinct field distributions of the two devices, as the size of the middle layer increases, as presented in Figures S5(b) and S5(d). Therefore, this structure can have a direct impact on traditional building blocks of photonic devices, such for example, lasers, and modulators. In Fig. S6(b) we compare the confinement factors of the scale-invariant waveguide to a single waveguide, in which its core is composed of the middle-layer material. One can see that for small dimensions compared with the wavelength, the scale-invariant waveguide presents better performance than the single waveguide itself. This is due to the fact that the high-index layers act as an optical cavity by trapping the light inside the middle layers, as can be seen comparing the field distributions in Fig. S5(a) and S5(e).

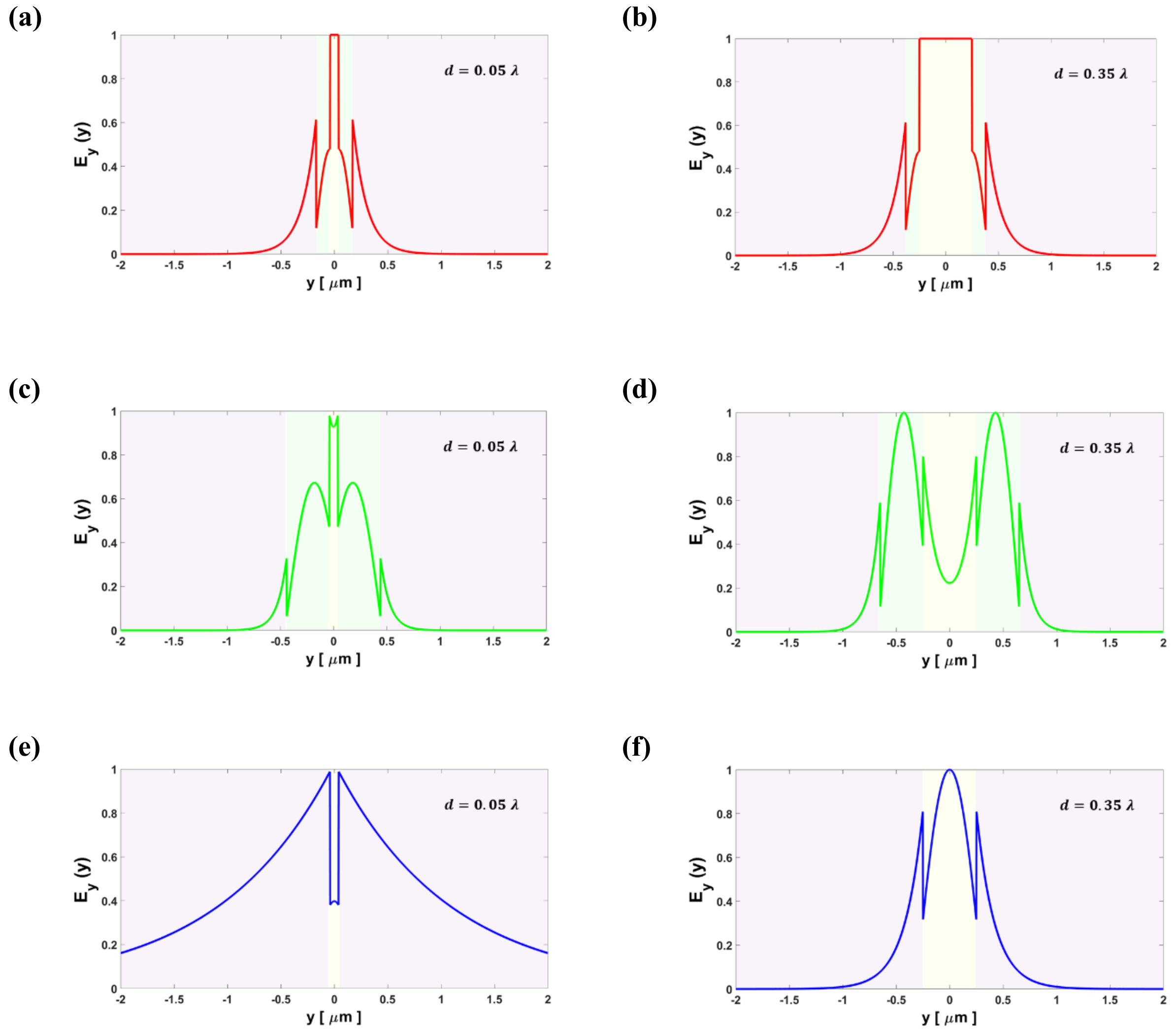

Figure S5. Field distributions $E_y(y)$ for the TM fundamental mode of distinct waveguide structures for two values of middle layer's thickness, $d = 0.05\,\lambda$, and $d = 0.35\,\lambda$. **(a-b)** Scale-invariant waveguide formed two high-index-material slabs $n_H = 3.5$ with thickness $t = 130\ nm$, separated by an intermediary-index material $n_S = 2.4212$ of thickness d, surrounded by a low-index-material $n_L = 1.5$. **(c-d)** Slot-waveguide with slabs thicknesses of $t = 400\ nm$. **(e-f)** Single slab waveguide composed of a core material with refractive-index $n_H = n_S = 2.4212$ and thickness $t = d$, surrounded by a low-index material $n_L = 1.5$. All the simulations are done assuming a wavelength of $\lambda_0 = 1.55\ \mu m$.

(a)

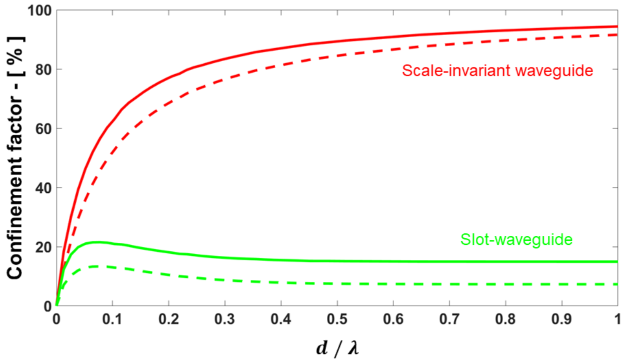

(b)

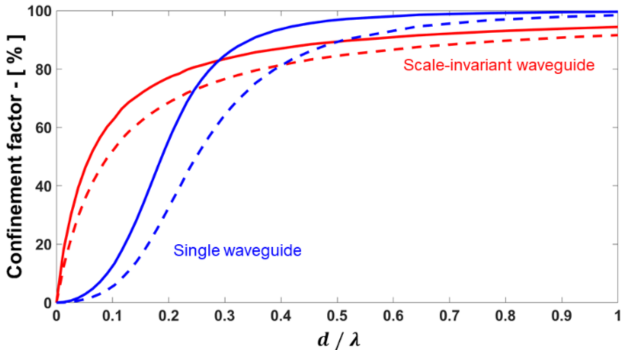

Figure S6. Confinement factors of the optical powers (solid lines) and Electric field intensities (dashed lines) inside the middle-layer region (n_S) as a function of its thickness d. Comparison between the scale-invariant waveguide and the slot-waveguide **(a)** and the single slab waveguide **(b)**. The parameters used in the simulations are the same described in Fig. S5.

4- Effect of material losses on the device

In this section, we analyze the effect of the material losses in the scale-invariant waveguide. We show that the proposed structure is robust against material losses for nominal loss values encountered in most dielectric materials in the Near-IR regime. Furthermore, we show how the effect can also be explored to leverage light confinement even with materials with high levels of gain/loss as in laser media or metallic materials. In order to show that, we consider the same dimensions and materials previously discussed ($n_H = 1.9954$, $n_L = 1.000$, $w/2 = 1\ \mu m$, $d = 5\ \mu m$, at $\lambda_0 = 1.55\ \mu m$ for the fundamental TM mode). We implemented this model in FDTD simulations by increasing the material losses (α) while keeping the real part of the refractive index (n) constant. Although the real part of the refractive index is intrinsically related to the imaginary part by the Kramers-Kronig (KK) relations, we assume that for each pair of n and α values it is possible to find a material that corresponds to these values at a given wavelength.

We divided our analysis into the following three cases: loss only in the intermediary material ($n_S + i\alpha$), loss only in the high-index material ($n_H + i\alpha$), and losses in both materials ($n_S + i\alpha$ and $n_H + i\alpha$). We consider the lowest-index material (n_L) lossless throughout these analyses. As discussed in the previous sections, the physics behind the proposed devices is based on getting a null transverse wavevector inside the intermediary material layers, $k_S = 0$, which is achieved by matching the intermediary material index with the mode effective index, i.e., $n_{eff} = n_S$. By now including material losses for a given material X, we are led to the definition of a complex refractive index for X, as $\widetilde{n_X} = n_X + i\alpha$, where n_X is the real part of the refractive index (Re ($\widetilde{n_X}$)) and α is the material absorption loss (Im ($\widetilde{n_X}$)). For low values of losses ($\alpha \ll n_X$), we have $\widetilde{n_X} \cong n_X$. Furthermore, since the wavevectors are functions of refractive index squared $\widetilde{n_X}^2$, low values of α are even less perceptive to the system. Figure S7(a) shows the real part of the effective index of the scale-invariant mode and its propagation loss in dB/cm, for all three cases. One can see for material losses $\alpha \leq 10^{-2}$, the mode effective index stays constant while the losses increase linearly on a logarithmic scale. This is the case for most dielectric materials currently in use in integrated photonics [2], where the loss values encountered in most of the foundries are well below 10 dB/cm ($\alpha < 3 \times 10^{-5}$) [2].

In the high-loss regime ($\alpha \sim n_X$), the effective index remains constant in the case of loss in both materials, the intermediary material ($\widetilde{n_S} = n_S + i\alpha$) and the high-index material ($\widetilde{n_H} = n_H +$

$i\alpha$), but it decays if only one of the materials is lossy. It is interesting to note that for the case of loss only in the high index material, $\widetilde{n_H} = n_H + i\alpha$, the propagation loss goes down due to an increase in the confinement of light in the intermediary material. Figure S7(b) shows the confinement factor for all three cases, in which one can see that the confinement factor not only stays constant for low values of losses but increases for high values of losses, making an even stronger case for the application of the proposed device in some metals and gain media.

(a)

(b)

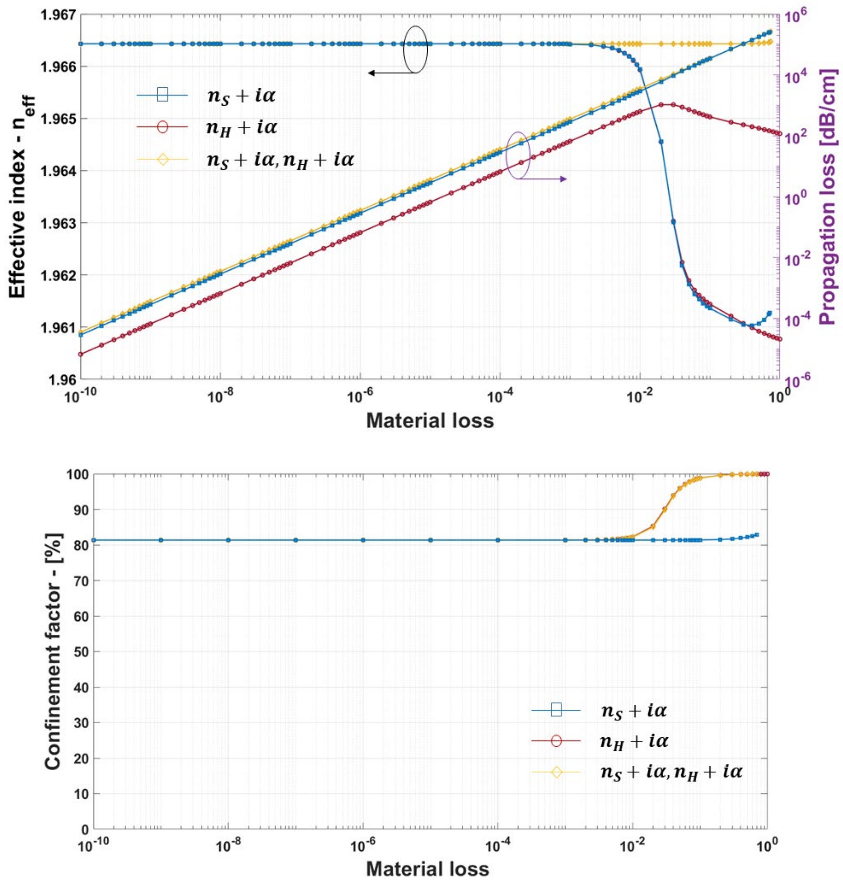

Figure S7. Effect of the material losses on the scale-invariant waveguide. **(a)** The real part of the effective indices and propagation losses for the scale-invariant mode as a function of the material loss considering loss only in the intermediary material (solid blue square lines), loss only in the high-index material (red circle line), and losses in both materials (yellow diamond line). **(b)** Confinement factor as a function of the material loss for all cases.

By using our MZI's experimental data and applying the approach described in [3], we estimated that our propagation losses are between 0.5 to 2 dB/cm. This relatively high loss is believed to be due to surface roughness and material absorption in SiON film. However, as previously described, this level of loss is not enough to perturb the scale-invariant mode.

5- Analytical-based method to design the 2D devices

As previously discussed, for a slab (planar) waveguide or 1D structure the critical point happens when the refractive index of the middle layer is equal to the effective index of the mode of a single waveguide without this layer, i.e., $n_{eff} = n_S$. However, for 2D geometric structures, the critical point happens for values slightly below that, i.e., $n_{eff} < n_S$, due to the vectorial nature of the electromagnetic field distribution in these 2D structures. We have developed a design method that still employs slab structures (and its semi-analytical solutions) to design these 2D waveguides.

Our design method is described by using the example illustrated in Fig. S8. In contrast to the main manuscript, here we consider a horizontally-oriented structure to show the versatility of the proposed device and its design method. The waveguide consists of two SiN materials separated by Al_2O_3 layer, deposited on a SiO_2 substrate and cladded with air (see Fig. S8(a)). In other to achieve the critical point in this structure, we calculate the n_{eff_slab} of the fundamental TE mode of an asymmetric slab waveguide composed of a core of Al_2O_3, a substrate of SiO_2, and air cladded, illustrated by the black dashed line in Fig. S8(a) and represented in Fig. S8(b). Next, we design the SiN strip waveguide width (w), shown in Fig. S8(c)), in order to match the effective index of its fundamental mode, n_{eff_strip}, with the effective index of the slab ($n_{eff\_strip} = n_{eff\_slab}$). This width is then used to achieve the scale invariance of the optical mode of the whole structure.

For this specific simulation, we consider the following properties for SiN, Al_2O_3, SiO_2, and air the indices are 1.9954, 1.7462, 1.4444, and 1.000, respectively. The waveguide thickness (t) is 730 nm and the wavelength of 1.550 nm. The effective index of the asymmetric slab mode n_{eff_slab} for the TE is calculated to be 1.6188. The strip SiN waveguide's width (w), in order for its Quasi-TE mode to match the slab effective index, is computed to be 376 nm. The simulation results for the previous example are shown in Figure S8.

The reasoning behind the design method it's that a slab waveguide assumes that the electrometric field is invariant in one direction (the direction assumed as infinity), and that is exactly what the uniform field distribution of the scale-invariant waveguide presents, at the critical point. We also notice that for SiN width lower than the desired one or larger wavelength, more light will be confined in Al_2O_3 materials, which enhances the interaction even more. Furthermore, we stress that the materials, polarization, and wavelength used here are merely illustrative and this

design technique can be employed in other structures. We use this method to design the device demonstrated in the main manuscript.

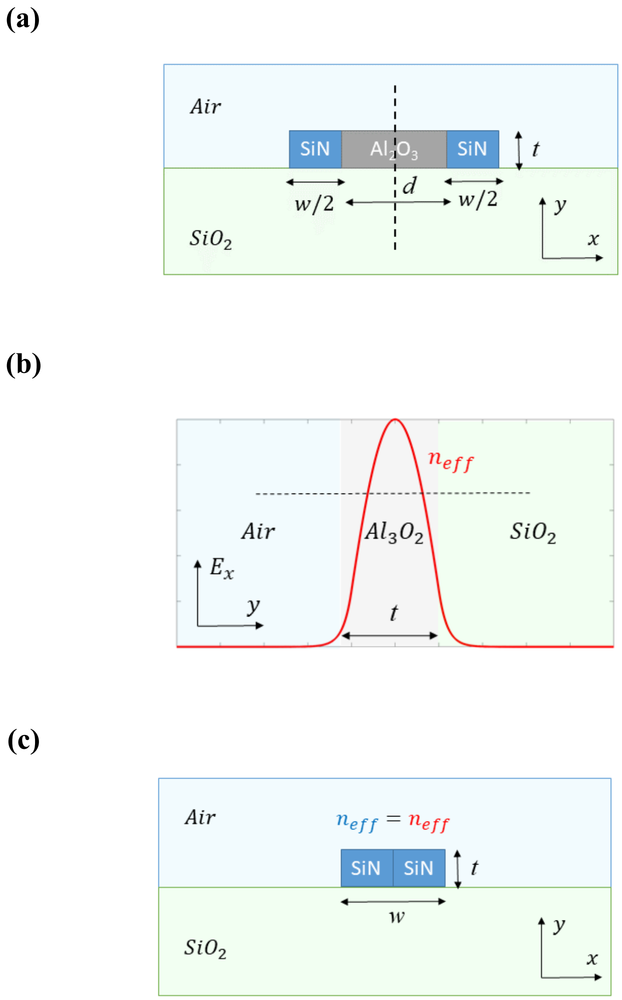

Figure S8. Designing the critical point in a 2D waveguide example. (a) Example of a 2D waveguide structure composed of two SiN waveguides separated by a middle layer of Al_2O_3, over a SiO_2 substrate and air cladded. **(b)** Slab waveguide formed by an Al_3O_2 core, SiO_2 substrate, and air cladded. Field distribution and effective index of the slab waveguide mode. **(c)** SiN strip waveguide is designed in such a way its Quasi-TE fundamental mode's effective index matches the effective index of the slab waveguide ($n_{eff\_strip} = n_{eff\_slab}$).

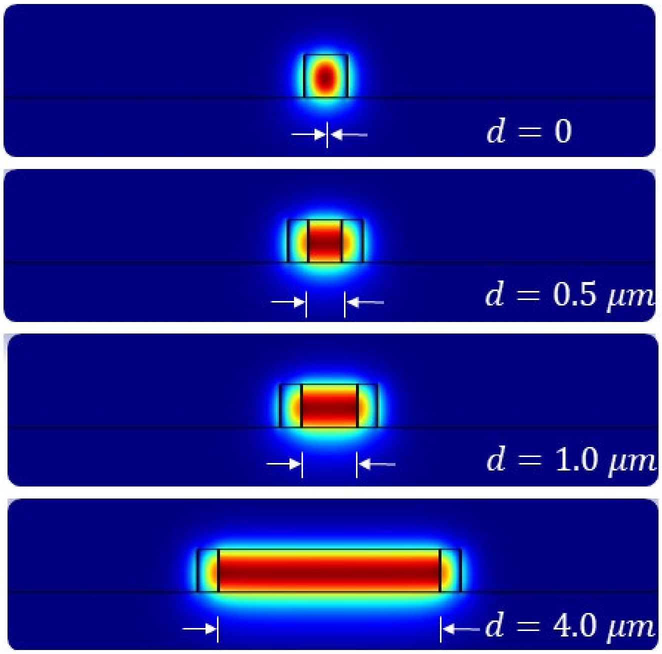

Figure S9. Numerical simulations of the structure at the critical point. Field distribution of fundamental Quasi-TE mode of the waveguide at the critical point for different values of the $d = 0$, 0.5, 2.0, and 4.0 μm.

6- Scale-invariant effect in low-refractive-index contrast waveguides

In this section, we analyze the scale-invariant effect in low-index contrast waveguides. The results presented in the main manuscript, including the experimental data, and the previous sections of the supplementary material were obtained using a relatively high-index contrast between SiN ($n_H \cong 1.99$) and SiO2 ($n_L \cong 1.45$): $\Delta = (n_H{}^2 - n_L{}^2)/2n_H{}^2 = 23\%$. However, this effect is very general and does not depend on specific values of the indices other than the already mentioned relation $n_H > n_S > n_L$. This generality can also be seen through the transcendental equations (S12 and S13), which do not make any assumptions about the values of indices. To exemplify this point, we first consider a low-index contrast 1D structure composed of $n_H = 1.460$, $n_L = 1.440$, $w = 2\ \mu m$, and $d = 8\ \mu m$ at $\lambda_0 = 1.55\ \mu m$. In this case, the index contrast is $\Delta = 1.36\%$ and therefore the structure is in the weakly guiding regime. Figures S10(a) and (b) show the field distribution for the TE and TM polarization and their non-Gaussian field distributions. Further, we consider a 2D waveguide () with the same index contrast ($\Delta = 1.36\%$), as shown in Fig. S11(a). The device parameters and dimensions are: $n_H = 1.4600, n_S = 1.4500, n_L = 1.4400, w/2 = 1.18\ \mu m, d = 8\ \mu m$, and $t = 5\ \mu m$ at $\lambda_0 = 1.55\ \mu m$. In order to get the scale-invariant effect we have followed the same designed method described in section 5. The FDTD simulations for the TE and TM polarizations are presented in Figures S11(b) and S11(c). One can see the non-Gaussian field distributions with a uniform profile is independent of the index contrast. Furthermore, due to the low-index contrast, the Quasi-TE and Quasi-TM have almost the same field profile distributions.

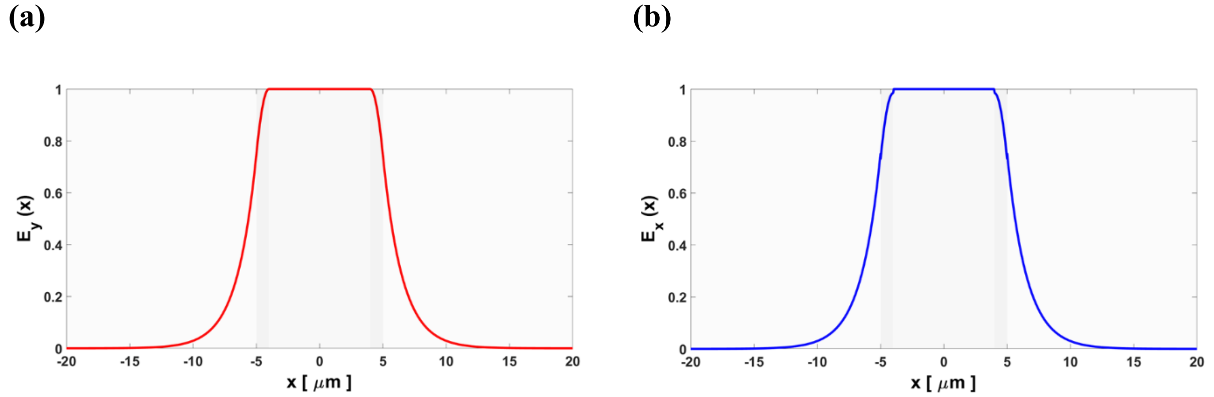

Figure S10. Scale-invariant effect in a 1D low-index-contrast waveguide. 1D simulation for the mode electric field profile distributions for **(a)** TE and **(b)** TM polarization for $\Delta = 1.36\%$ index contrast. All simulations are done at 1550 nm.

(a)

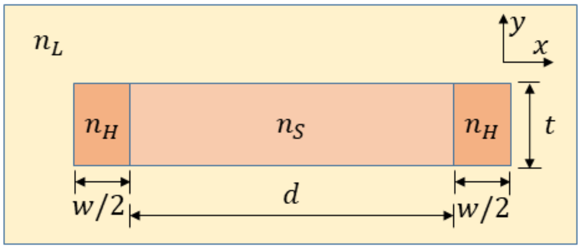

(b)

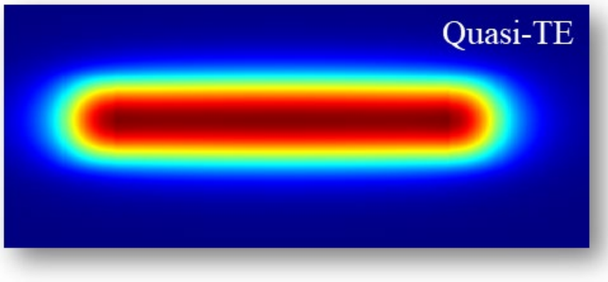

(c)

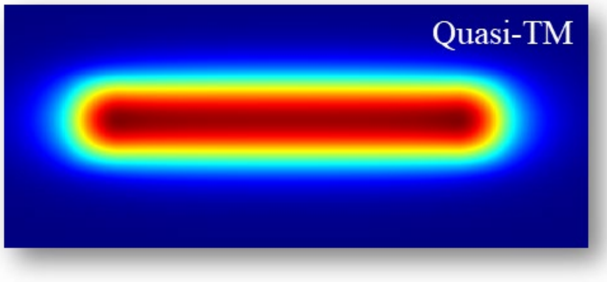

Figure S11. Scale-invariant effect in a 2D low-index-contrast waveguide. **(a)** Schematics of the geometric structure for the scale invariant waveguide. 2D FDTD simulations of the electric field intensity profiles for the **(b)** Quasi-TE and **(c)** Quasi-TM polarizations, for a $\Delta = 1.36\%$ index contrast at 1550 nm.

7- Materials characterization and the waveguide design

The materials were characterized with a variable-angle spectroscopic ellipsometer (J. A. Woollam VASE). Figure S12 shows the refractive index of each material and the fitting. The refractive indices for each material at 1.55 μm are for SiN is $n_{SiN} = 1.9954$, Al2O3 is $n_{Al_3O_2} = 1.7565$, and SiO2 is $n_{SiO_2} = 1.4444$.

(a)

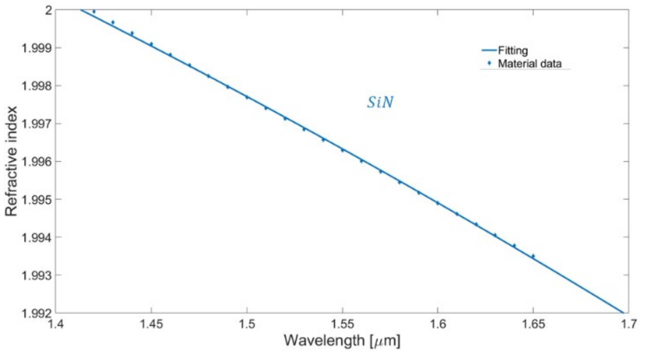

(b)

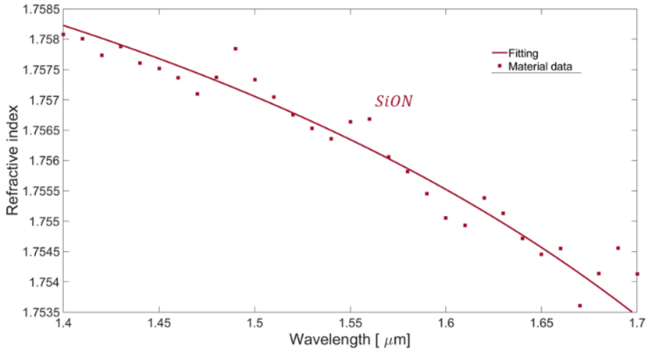

(c)

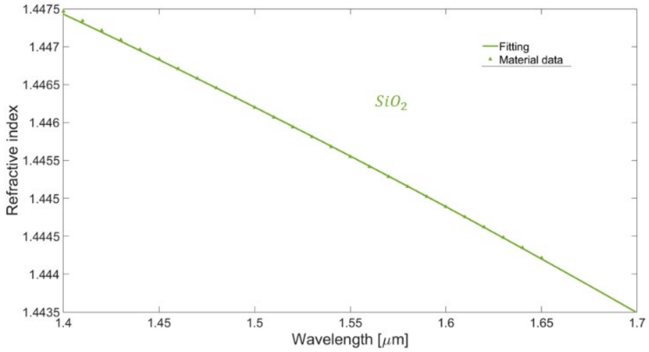

Figure S12. Materials refractive indices and fitting. Refractive indices and fitting for SiN **(a),** SiON **(b),** and SiO_2 **(c)**.

We design the scale-invariant waveguide to operate at the critical point at 1.55 μm wavelength. By following the designing approach described previously, we defined the waveguide width to be $w = 1\ \mu m$. Then, we simulated a symmetric slab defined by 1 μm-SiON core and SiO_2 as substrate and cladding. The effective index of the designed slab is calculated to be $n_{eff\_slab} = 1.6641\ \mu m$ for its fundamental TM mode (horizontally-oriented slab), as shown in Figs. S13. After that, we simulate a strip SiN waveguide and variate its thickness in order to make its fundamental quasi-TE mode matches the slab effective index ($n_{eff\_strip} = n_{eff\_slab}$). The computed thickness is $t_{SiN} = 452$ nm.

(a)

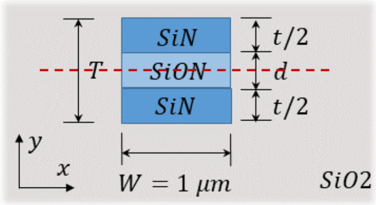

(b)

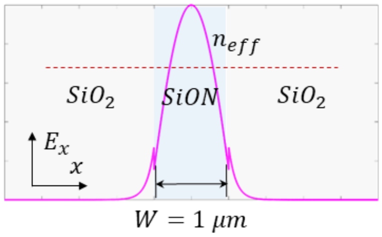

(c)

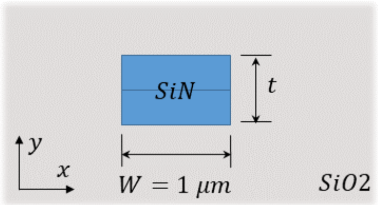

Figure S13. Designing the critical point scale-invariant waveguide. (a) Scale-invariant waveguide composed of two SiN layers separated by SiON middle layer, and cladded by SiO_2. **(b)** Slab waveguide formed by a SiON core and a SiO_2 clad. Field distribution and effective index of TM fundamental mode. **(c)** SiN strip waveguide is designed in such a way that its quasi-TE fundamental mode's effective index matches the effective index of the slab waveguide ($n_{eff\_strip} = n_{eff\_slab}$).

8- Analysis of the operation bandwidth of the device

The scale-invariant effect, meaning the effect of increasing the size of the waveguide while keeping the mode distribution and its effective index constant, only happens at a single frequency (wavelength) for a given structure and, therefore, can be classified as a narrow-band effect. This can be seen through the transcendental equations, where the effective index of the mode is computed for a single frequency (${k_S}^2(\lambda_0) = 0$), or through the ray-optics picture, where the critical angle between two dielectric interfaces is unique ($\theta_{inc}(\lambda_0) = \theta_{crict}(\lambda_0)$) for a given wavelength. More generally, these exceptional points are singularities that only happen at special (critical) points on the parameter space. As presented in the main manuscript, we designed our device in order to demonstrate this effect exactly at 1550 nm. However, here we show that the property that we are exploring in this waveguide, i.e., the enhancement of the field overlap with the intermediary-index (n_S) material, is very broadband. The reason for this broadband behavior is that a small variation around the critical point, $k_S = 0$, leads to only a small variation around the uniform distribution, $\left.{}^{\partial k_S}/_{\partial \lambda}\right|_{\lambda_0} \approx 0$. By considering the modulus of the wavevector, $|k_S|$, the critical point is the minimum of a symmetric square root function. For wavelengths larger than the critical wavelength ($\lambda > \lambda_0$) the mode leaks into the middle material creating a concave curvature. On the other hand, for wavelengths smaller than the critical wavelength ($\lambda < \lambda_0$) the mode concentrates more into the high-index material creating a convex curvature in the field distribution. However, for middle layer sizes (d) of the order of a few wavelengths or smaller, most of the light is still concentrated inside it. To exemplify this, first, we consider a 1D structure composed of $n_H = 2.0$, $n_L = 1.45$, $w/2 = 1\ \mu m$, and $d = 5\ \mu m$. The waveguide was designed to present the uniform field distribution at exactly $\lambda_0 = 1550\ nm.$ Figures S14(a) and S14(b) show the TE (E_y) and TM (E_x) mode field spatial distributions respectively at the critical point and $\pm\delta\lambda_0 = 50\ nm$ wavelength variation around this point. One can see that in all cases most of the light is still localized inside the middle layer. The electric field confinement factor for both polarizations is almost constant (with variation less than 2%) throughout and beyond an entire telecom band (C-band), as presented in Fig. S14(c).

(a)

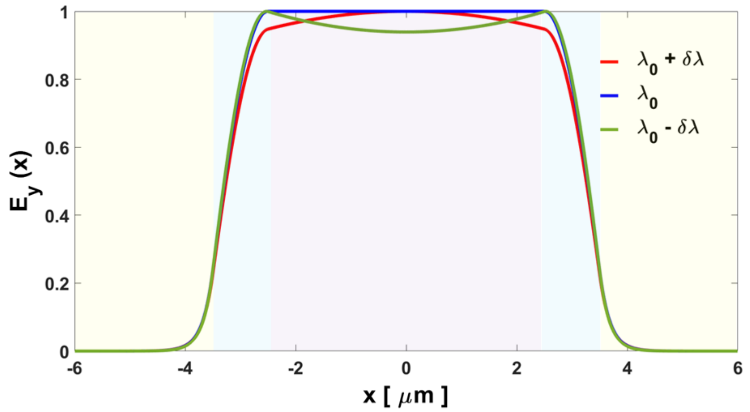

(b)

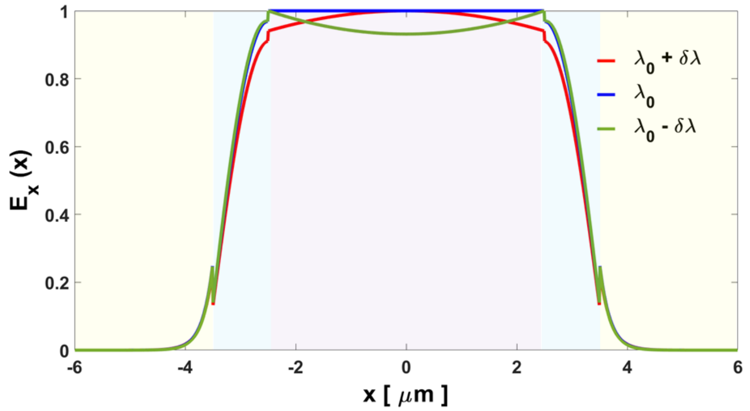

(c)

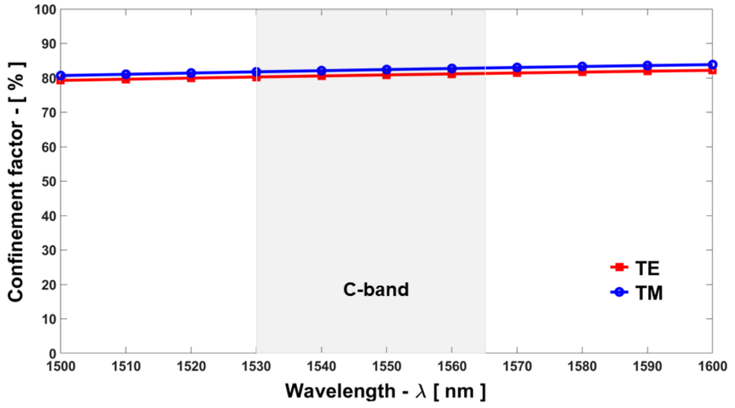

Figure S14. Operation bandwidth of the 1D scale-invariant waveguide. 1D simulation for the mode electric field profile distribution for **(a)** TE and **(b)** TM polarization for three different values of wavelengths 1500, 1550, and 1600 nm. The device was designed to present the scale-invariant effect at the center wavelength ($\lambda_0 = 1550$ nm). **(c)** Electric field intensity confinement factor as a function of the wavelength for the TE and TM polarizations.

In the previous demonstrated example, we only considered the waveguide dispersion in a simplified 1D structure. Next, we considered the waveguide cross-section used in our experimental demonstration, including the dispersion of the materials presented in Section 7. Figure S15(a) shows the FDTD simulation of the electric field intensity distributions for three distinct values λ_0, 1500, 1550, and 1600 nm, in which one can see that most of the light is still concentrated in the middle material for all the cases. Thus, the variation of the field distribution with wavelength barely affects the confinement factor which remains nearly constant across the whole bandwidth of 100 nm (~ 12.5 THz), as is presented in Figure S15(b).

(a)

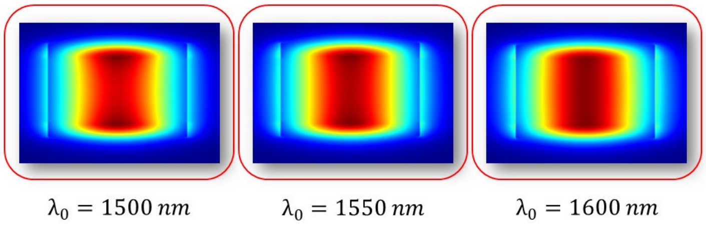

(b)

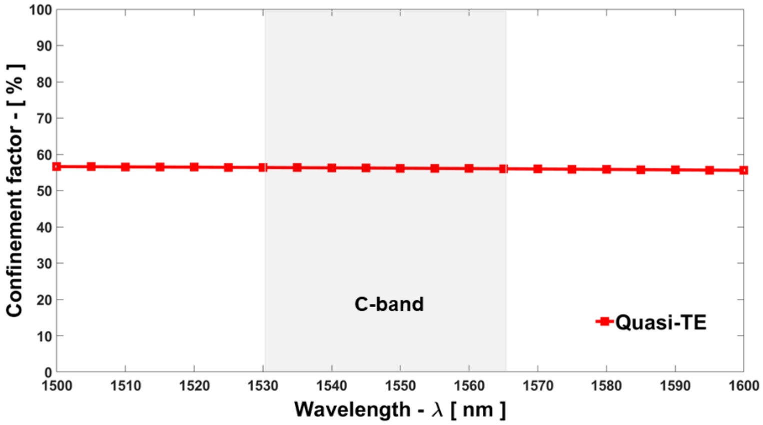

Figure S15. Operation bandwidth of 2D scale-invariant waveguide. **(a)** 2D FDTD simulations of electric field profile for the Quasi-TE polarization for three different values of wavelengths 1500, 1550, and 1600 nm. The device was also designed to present the scale-invariant effect at the center wavelength ($\lambda_0 = 1550$ nm). **(b)** Electric field intensity confinement factor as a function of the wavelength for the Quasi-TE polarization.

9- Effect of geometric imperfections on the device

Undesired variations in the microfabrication processes introduce imperfections in the waveguide dimensions and, therefore, can affect the mode of the scale-scale invariant waveguide. For vertically-oriented stacks, the variations are less perceptive since in most deposition techniques the film thickness can be well controlled (down to a few nanometers), through careful pre-characterization of their deposition rates. On the other hand, for horizontally-oriented stacks, there are differences between the layout mask's nominal widths and the fabricated dimensions. Nowadays, these variations are around $\pm 10\ nm$ as described in the PDK's (Process Design Kit's) of most photonics foundries [2]. Here we show that, although this difference can be enough to take the scale-invariant waveguide out of the critical point, it does not significantly affect the electric field intensity concentration inside the middle layer. Furthermore, we show that a symmetric variation creates the same effect as the previously described wavelength variation, while an asymmetric variation creates an inclination in field distribution. We show that in all the cases, the confinement factor stays practically constant proving the robustness of the proposed structure.

For symmetric variations in the high-index-material layer' width, $w/2 \pm \delta\, w/2$, meaning the same variation in both high-index-material layers, the behavior is exactly the same as the wavelength variation. The reason for that is the fundamental intrinsic relation between the high-index core dimension, w, and the light wavelength, λ_0, in a dielectric slab waveguide, $-\lambda_0 \left.{}^{dn_{eff}}\!/_{d\lambda}\right|_{w} = w \left.{}^{dn_{eff}}\!/_{dw}\right|_{\lambda_0}$. For smaller widths ($w/2 - \delta\, w/2$), the mode leaks more to the middle material creating a convex curvature, while for larger widths ($w/2 + \delta\, w/2$) the mode concentrates more inside the high-index-material layers creating a concave curvature. The minus sign in the width-wavelength relation shows thinner layers have the same effect as a larger wavelength, and vice-versa. Therefore, the critical point happens for smaller wavelengths for a smaller layer's widths. Besides that, the conclusions are equivalent to the bandwidth analysis, where in all the cases most of the light is still concentrated inside the middle layer. Figures S16(a) and S16(b) show the mode profile distributions for the TE and TM polarization, respectively, considering the device parameters used in the bandwidth analysis but considering variations in the high-index-layer widths $\pm\delta\, w/2$ ($\pm\, 10\, nm$) at a fixed wavelength $\lambda_0 = 1550\, nm.$ For asymmetric variations where one side of the high-index-materials layer is larger (or smaller) than

the other, an inclination in the field distribution is created. The reason for that can be understood from the Supplementary equations S10 and S12, which show that the full solution is a linear distribution for operation near the critical point. It is worth noting that the field inclination can be controlled by designing the waveguide asymmetry ($w/2 - \delta w/2$ and $w/2 - 2\delta w/2$), as presented in Fig. S16(c) and S16(d), which can find practical applications by itself.

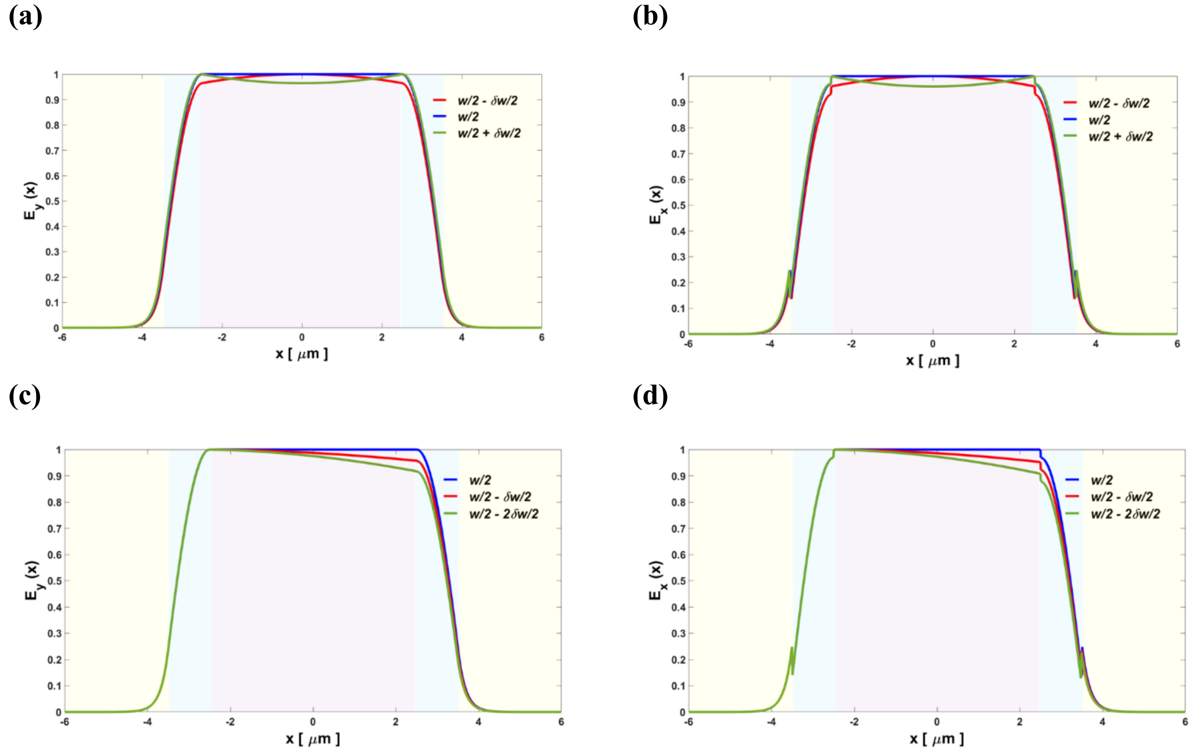

Figure S16. Effect of geometric imperfections on the 1D scale-invariant waveguide. 1D simulations of the electric field profile distribution for **(a)** TE and **(b)** TM polarizations, respectively, for symmetric variations around the designed high-index layer's widths ($w/2 \pm \delta w/2$). **(c)** TE and **(d)** TM polarizations, respectively, for asymmetric variations ($w/2 - \delta w/2$ and $w/2 - 2\delta w/2$) only in the right-side high-index layer's width. All simulations were done at a fixed wavelength $\lambda_0 = 1550$ nm.

In order to analyze the effect of the dimension imperfections in a real structure, we consider the 2D waveguide cross-section ($n_H = 2.0$, $n_L = 1.45$, $w/2 = 0.337\ \mu m$, $t = 1\ \mu m$, and $d = 2\ \mu m$), as described in Figure S51(a), at the wavelength 1550 nm, and consider dimensional variations in the high-index material's width $\pm\delta w/2$ ($\pm$ 10 nm). Figure S17 shows the FDTD

simulation of the electric field intensity distributions in all cases described for the 1D structure and the confinement factor inside the middle layer for each case. One can see that besides presenting the same behavior as the 1D structure, the confinement factor values show that most of the light is still concentrated in the middle material for all the cases.

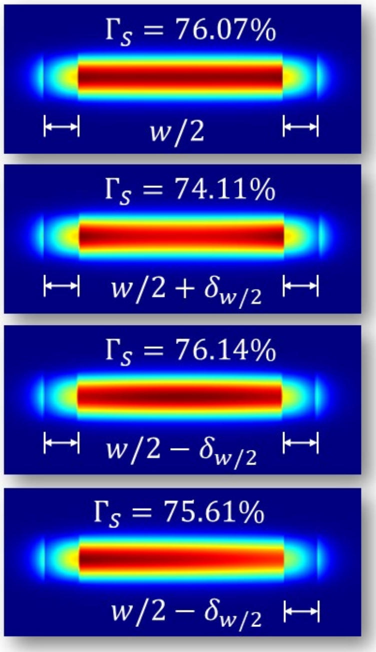

Figure S17. Effect of geometric imperfections on the 2D scale-invariant waveguide. 2D FDTD simulations of electric field profile for the Quasi-TE polarization for the designed width, for a symmetric variation created by larger widths ($w/2 + \delta\, w/2$) on both sides, a symmetric variation created by for a smaller width ($w/2 - \delta\, w/2$) in both sides, and for an asymmetric variation with smaller width just in the right side. The electric field intensity confinement factors in the middle layer are described for all the cases. All simulations were done considering a fixed wavelength at $\lambda_0 = 1550$ nm.

10- Lensed-fiber pre-characterization

We controlled the displacement of the input and output fibers by using a high-resolution piezo-stage (NanoMax from Thorlabs). We used a lensed-nanofiber TMSJ-3A-1550-9 (from OZOptics) with a nominal spot diameter of 2.5 μm at a working distance of 14 μm. We pre-characterize the mode profile of the input lensed-fiber by vertically translating a second (output) lensed-fiber and recording the output signal in a power meter at a working distance of 20 μm, as shown in Fig. S18. We recovered the input lensed-fiber mode profile by using a Matlab-based deconvolution algorithm and the convolution properties of Gaussians functions (reference). The fiber mode profile was then used to recover the modes images presented in the main manuscript.

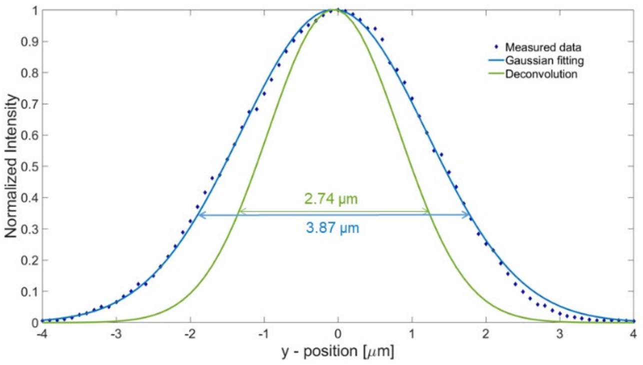

Figure S18. Pre-characterization of the lensed nanofiber. Measured data and Gaussian fitting of the convolution of the input and output fibers. Deconvolution of the experimental data.

Supplementary References

[1] Robinson, J. T., Preston, K., Painter, O., and Lipson M, "First-principle derivation of gain in high-index-contrast waveguides," Opt. Express 16, pp. 16659-16669 (2008).

[2] Siew S. Y et al., "Review of Silicon Photonics Technology and Platform Development," in Journal of Lightwave Technology, vol. 39, no. 13, pp. 4374-4389 (2021).

[3] Gutierrez, A. M., Brimont, A., Aamer, M., and Sanchis, P., "Method for measuring waveguide propagation losses by means of a Mach–Zehnder Interferometer structure," Optics Communications, vol. 285, no. 6, pp. 1144-1147 (2012).
